# A High Performance LIA-Based Interface for Battery Powered Sensing Devices

**DOI:** 10.3390/s151025260

**Published:** 2015-09-30

**Authors:** Daniel García-Romeo, María R. Valero, Nicolás Medrano, Belén Calvo, Santiago Celma

**Affiliations:** Group of Electronic Design, Aragon Institute for Engineering Research, I3A, Facultad de Ciencias, Pedro Cerbuna 12, Zaragoza 50009, Spain; E-Mails: mrvalero@unizar.es (M.R.V.); nmedrano@unizar.es (N.M.); becalvo@unizar.es (B.C.); scelma@unizar.es (S.C.)

**Keywords:** CMOS analog integrated circuits, portable lock-in amplifiers, low-voltage low power, gas sensing

## Abstract

This paper proposes a battery-compatible electronic interface based on a general purpose lock-in amplifier (LIA) capable of recovering input signals up to the MHz range. The core is a novel ASIC fabricated in 1.8 V 0.18 µm CMOS technology, which contains a dual-phase analog lock-in amplifier consisting of carefully designed building blocks to allow configurability over a wide frequency range while maintaining low power consumption. It operates using square input signals. Hence, for battery-operated microcontrolled systems, where square reference and exciting signals can be generated by the embedded microcontroller, the system benefits from intrinsic advantages such as simplicity, versatility and reduction in power and size. Experimental results confirm the signal recovery capability with signal-to-noise power ratios down to −39 dB with relative errors below 0.07% up to 1 MHz. Furthermore, the system has been successfully tested measuring the response of a microcantilever-based resonant sensor, achieving similar results with better power-bandwidth trade-off compared to other LIAs based on commercial off-the-shelf (COTS) components and commercial LIA equipment.

## 1. Introduction

Cyber physical systems (CPS) embody one of the major driving forces that go beyond the cyber world toward the physical world. Current embedding technologies have played key roles to enable rich interactions with the physical environment in nearly invisible ways [1].

Applications of CPS include medical devices and systems; assisted living; traffic control and safety; advanced automotive systems; process control; energy conservation; environmental control; transport; instrumentation; infrastructure control; distributed robotics; security; defense; manufacturing and smart structures [2,3]. Furthermore, new capabilities can be envisioned as well, such embedded intelligence in automobiles to improve safety and efficiency in transportation, building control systems to improve energy efficiency and so on, all of them with huge economic impact [4,5].

CPS typically consists of sensor and/or actuator networks integrated under an intelligent decision system. They bridge the physical world to the cyber world through sensors and actuators. Hence, a critical element is the electronic interface that conditions the sensing outputs into signals to be processed into embedded computers like microcontrollers (µC) [6].

In the energy-constrained scenario of portable systems in CPS, ensuring the correct acquisition of sensor data in high noise environments is an ever-increasing challenging task. With the reduction of power supply voltage, electronic interfaces for CPS are losing a significant amount of operating range. As a result, the sensor output signals can turn out to be very small compared to the noise level. In these cases, specific techniques for extracting the signal information should be considered.

An interesting possibility is the use of lock-in amplifiers (LIAs) [7], which use the phase sensitive detection (PSD) [8] technique to single out the data signal at a specific reference frequency rejecting noise signals at other frequencies. Digital PSD implementations [9,10] are appropriate when the processing electronics includes a processor with enough computing power to undertake the mathematical operations. However, for battery-fed embedded sensing systems, which include low cost microcontrollers with limited computing power, analog LIAs become a most suitable option [11,12,13,14,15,16,17].

Although widely employed in instrumentation, only a few analog integrated LIAs can be found in the literature. Most of the previous integrated solutions present an operating frequency below 50 kHz [14,15,16,17]. This value may be too low for some applications, like in density-viscosity sensors based on piezoelectric MEMS resonator and oscillator circuits, where resonant frequencies higher than 300 kHz are commonly used [18], or explosives detection where resonant sensors work at 60–80 kHz [19]. Moreover, a higher bandwidth (BW) not only increases the range of signals which can be processed but the maximum speed rate at which measurements can be taken too [20].

Most analog integrated LIAs use a sinusoidal wave as system input [21]. Nevertheless, in microcontrolled systems, a more compact, low-voltage, and low-power solution can be reached by considering square ones [22]. Conversely, square-signal processing imposes high demands in the design of the LIA and its main blocks, especially in terms of slew rate and bandwidth. Therefore, their design needs to be optimized for a good trade-off between power and performance. In response to the above, this paper presents a LIA-based electronic general-purpose interface able to recover square input signals with frequencies as high as 1 MHz and buried in high noise levels. The core of the system is a novel ASIC fabricated in 1.8 V-0.18 µm CMOS technology.

In addition to the CMOS LIA-based signal conditioning electronics, the proposed interface contains on a single PCB a digitation stage and a µC, thus allowing a full signal and information processing. Its aim is to provide a versatile, low-cost, low-power solution for multi-sensing and/or embedded sensing applications in CPS.

This paper is organized as follows: Section 2 describes the interface design, whereas Section 3 reports its experimental characterization. Section 4 shows its application to microcantilever-based sensors for gas sensing purposes. Finally, conclusions are drawn in Section 5.

## 2. Interface Design

**Figure 1:**
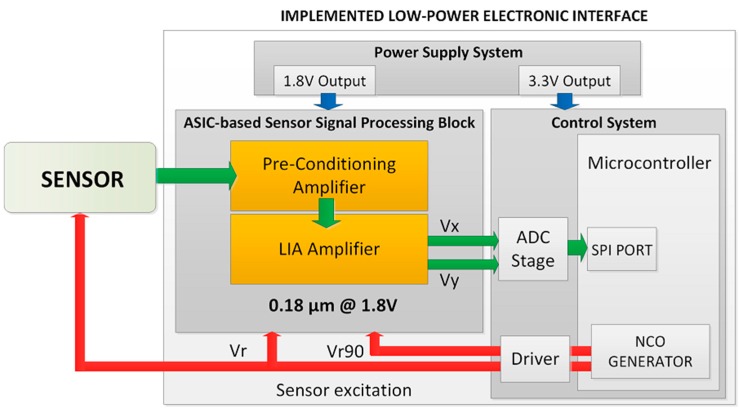
Electronic interface for portable sensing applications.

Figure 1 shows the block diagram of the proposed electronic interface, mainly composed by three blocks: an ASIC-based sensor signal processing, comprising a pre-conditioning amplifier and an analog LIA; a control system, based on a high performance low-cost low-power microcontroller; and a power supply system that provides the suitable voltage levels to every interface component from a battery.

### 2.1. Sensor Signal Processing System

**Figure 2:**
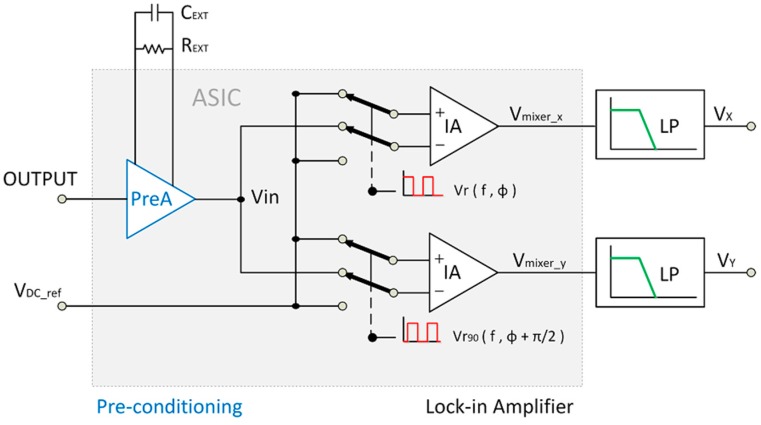
Block diagram of the signal conditioning stage.

Figure 2 shows the block diagram of the custom sensor signal processing system. It has been designed in a low cost 1.8 V-0.18 µm CMOS technology. The system design is fully compatible with digital technologies, thus allowing its implementation in future mixed-mode full-custom System-on-Chip (SoC) solutions. The signal processing system consists of a pre-conditioning amplifier and a dual-phase LIA.

The pre-conditioning amplifier, based on a classical inverting operational amplifiers (OpAmp)-based scheme, provides high input impedance, sets the adequate common-mode voltage to *V_DC_ref_* = *V_DD_*/2 for a maximum input range under single *V_DD_* supply operation and amplifies the sensor *OUTPUT* signal by a gain factor G selectable through an external feedback resistor R_EXT_. Besides, the maximum bandwidth of this input stage can be reduced by means of an external capacitor C_EXT_ in parallel with R_EXT_ to reduce noise contribution.

Next, the integrated dual-phase LIA recovers the information from the preconditioned sensor signal *V_in_*. The choice of a dual-phase architecture avoids phase dependency [11,12]. Each of the two parallel PSD branches includes an instrumentation amplifier (IA) with switched inputs, which are respectively controlled by two square signals *V_r_* and *V*_*r*90_, with 90° phase shift, so that the PSD or mixer output is:

(1) $$V_{mixer\_x,y}=\{\begin{array}{c} \left(V_{in}-V_{DC\_ref}\right)\text{ if }V_{r,r90}>V_{DD}/2\\ \left(V_{DC\_ref}-V_{in}\right)\text{ if }V_{r,r90}<V_{DD}/2 \end{array}$$

In this way, *V_in_* is rectified over the virtual ground *V_DC_ref_* = *V_DD_*/2. Then *V_mixer_x_*, *V_mixer_y_* are low-pass filtered providing the mean values *V_X_*, *V_Y_*. An external selectable first order R_L_C_L_ low pass filter has been chosen for high versatility. For the tests next conducted, the low pass filter cut-off frequency has been set to 5 Hz, as a trade-off between low pass filtering and measurement speed.

Finally, for signal information recovery, if *V_in_* is a sinusoidal signal, its amplitude *A* and phase φ can be recovered from *V_X_* and *V_Y_* as indicated in [11]. Likewise, if *V_in_* is a square signal, then signal amplitude and phase φ can be obtained as indicated in Table 1 [22].

**Table 1:** Amplitude and phase recovery equations for an input square wave.

*V_X_*	*V_Y_*	*A*	*φ*
$<\frac{V_{DD}}{2}$	$<\frac{V_{DD}}{2}$	$V_{DD}-V_{X}-\;V_{Y}$	$\left(\frac{V_{Y}-\frac{V_{DD}}{2}}{V_{X}+V_{Y}-V_{DD}}\right)\frac{\text{π}}{2}\;\in \left(0,\frac{\text{π}}{2}\right)$
$>\frac{V_{DD}}{2}$	$<\frac{V_{DD}}{2}$	$V_{X}-\;V_{Y}$	$\text{π}+\;\left(\frac{V_{Y}-\frac{V_{DD}}{2}}{V_{X}-V_{Y}}\right)\frac{\text{π}}{2}\in \;\left(\frac{\text{π}}{2},\text{π}\right)$
$>\frac{V_{DD}}{2}$	$>\frac{V_{DD}}{2}$	$V_{X}+\;V_{Y}-\;V_{DD}$	$\text{π}+\;\left(\frac{V_{Y}-\frac{V_{DD}}{2}}{V_{Y}+V_{X}-V_{DD}}\right)\frac{\text{π}}{2}\in \;\left(\text{π},\frac{3\text{π}}{2}\right)$
$<\frac{V_{DD}}{2}$	$>\frac{V_{DD}}{2}$	$V_{Y}-\;V_{X}$	$2\text{π}-\left(\frac{V_{Y}-\frac{V_{DD}}{2}}{V_{Y}-V_{X}}\right)\frac{\text{π}}{2}\;\in \left(\frac{3\text{π}}{2},2\text{π}\right)$

**Figure 3:**
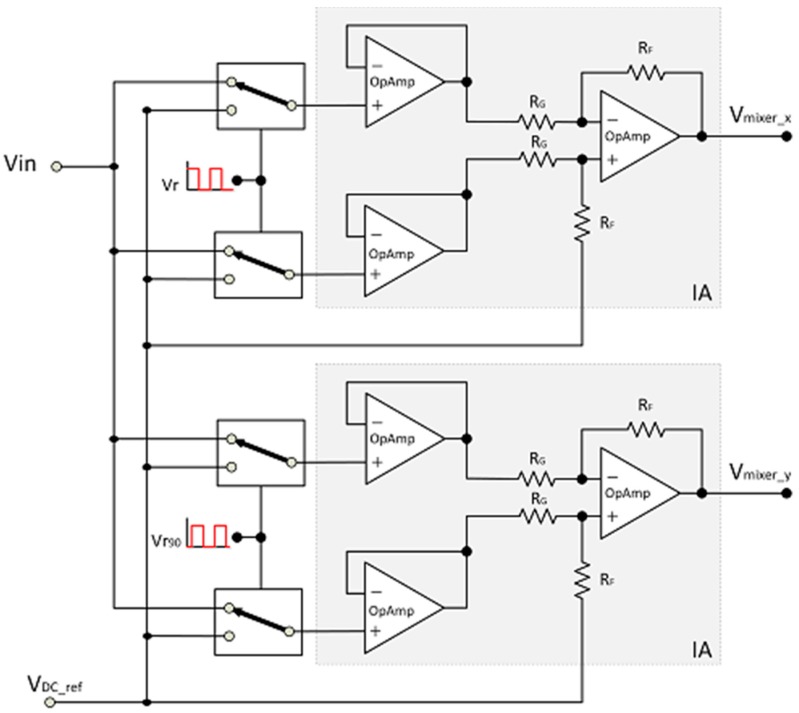
LIA scheme, detailing the instrumentation amplifier architecture: a precision differential architecture design with input buffering.

Figure 3 details the scheme of the dual LIA. A precision differential amplifier architecture with input buffering has been chosen as instrumentation amplifier. Resistors R_F_ and R_G_ have been implemented with a value of 10 kΩ, thus setting the gain of this stage to 1. The IA active building blocks are operational amplifiers, all identical and equal to the OpAmp used in the pre-conditioning stage for modularity. Besides, this unity-gain design makes that the dynamic performances of the IA are straightforward determined by those of the core OpAmp, which is thus a critical device. Due to the lowering of power and supply voltage in portable applications, OpAmps lose a significant amount of operating range. These constraints impose special demands on their low-voltage low-power (LVLP) high performance CMOS design [20].

In our case, rail-to-rail input/output operation maximizes the input signal span and increases the signal-to-noise ratio (SNR). It is also necessary to guarantee that the signal is exactly rectified at the midpoint of the supply voltage range (*V_DD_*/2). Class AB operation improves power-efficiency, but it also allows fast settling response and high slew rate. This reduces phase jitter between the input and the processed signals and allows processing square signals, as those provided in microcontrolled-based systems [23]. Other performances, such as high common-mode rejection ratio (CMRR), low distortion, high power supply rejection ratio (PSRR) and bandwidth (BW) are also to be preserved.

**Figure 4:**
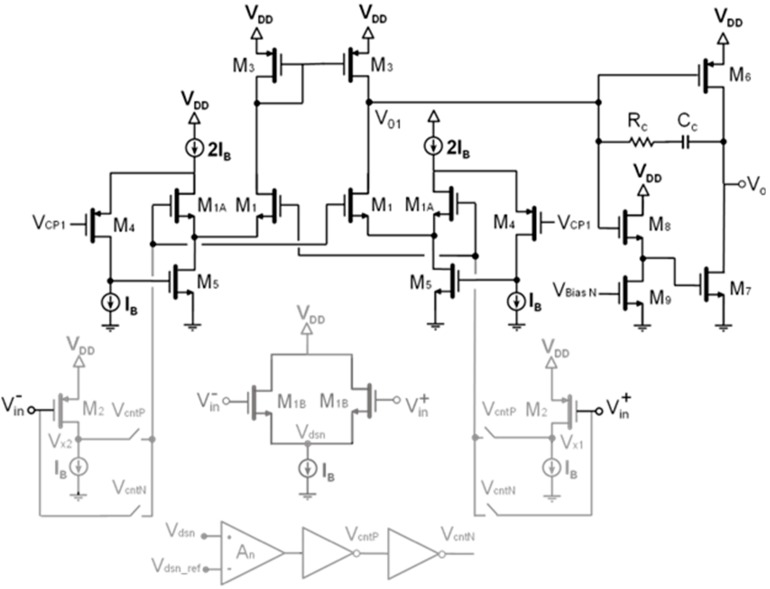
Schematic of the designed 1.8 V class AB rail-to-rail two stage OpAmp. The auxiliary control circuit for rail-to-rail operation is depicted in grey.

Taking into consideration all the above, Figure 4 shows the schematic of the specifically designed two-stage AB OpAmp. Rail-to-rail common-mode input range is achieved by using a novel LVLP technique for rail-to-rail operation based on a MOS input stage with only one differential pair and switched level shifters driven by an auxiliary control circuit [24]. Further, the NMOS input pair M_1_ is class AB biased by means of adaptive biasing. The reason is twofold: firstly to boost the input pair currents when a large differential input signal *V_diff_* = *V_in+_* − *V_in−_* is applied, and secondly, to reduce the dependence of the input pair currents (and then, the transconductance) on the common mode voltage. In this case, cross-coupled floating batteries (M_1A_, M_4_, M_5_) have been used for adaptive biasing. A rail-to-rail push-pull output stage has been used for a class AB output [20]. The first stage output *V_01_* is directly tied to the PMOS transistor M_6_ of the rail-to-rail push-pull output block, and through a level shifter (M_8_, M_9_) in the complementary NMOS transistor M_7_ biased with an adequate DC level. Finally, the two stage OpAmp is compensated by means of a classical Miller compensation. Table 2 summarizes the main characteristics of this integrated 0.18 μm CMOS OpAmp supplied at a single 1.8 V.

**Table 2:** Operational amplifier performance summary.

**CMOS Process**	0.18 μm
**Supply**	1.8 V
**Open Loop Aain**	74 dB *
**Gain-Bandwidth Product**	2 MHz
**CMRR**	96 dB *
**Slew-Rate**	2.3 V/μs (for a capacitive load of 1 nF)
**THD**	−62 dB (1 kHz, 1.75 V_pp_)
**Input Common-Mode Range**	Rail-to-rail
**Output Swing**	Rail-to-rail
**Input Referred Noise**	63 nV/Hz^1/2^* (100 kHz)
**Power**	468 μW

* Simulation results.

**Figure 5:**
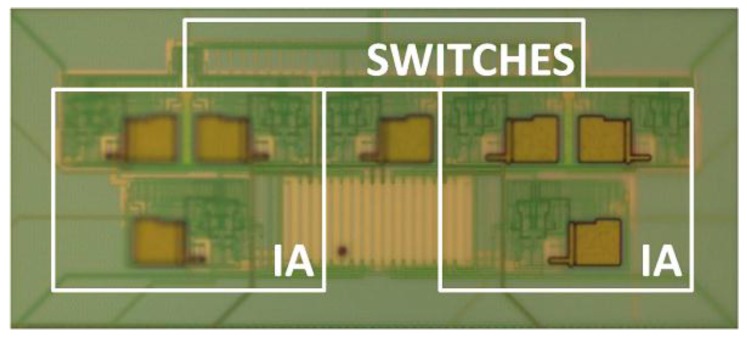
Integrated lock-in amplifier (detail) for the prototype, with an active area of 306 × 114 μm^2^. It includes the switches and instrumentation amplifiers (IA).

Figure 5 shows a microphotograph of the LIA active area (detail). The chip area and quiescent power consumption equal 306 × 157 μm^2^ and below 2 mW per branch respectively.

### 2.2. Control System

The processing interface is controlled by a P8X32A Propeller microcontroller from Parallax (Parallax Inc., Rocklin, CA, USA). This microcontroller has been selected due to its high-speed processing features while maintaining a low current consumption and a small physical footprint. In order to perform this high-speed processing it comprises eight modules or processors (cogs) that can operate simultaneously. All eight processors are controlled from the same internal system clock so they use the same time reference. Each processor contains two advanced numerically controlled oscillators (NCO) that allow a precise frequency selection and phase shift. This feature allows using the microcontroller to generate in each cog two accurate square signals in quadrature configuration *V_r_* and *V*_*r*90_ required by the LIA to perform the PSD measurements.

The capability of the microcontroller to generate several quadrature square signals is especially relevant interfacing sensors arrays, as for instance, the so called electronic noses [19,23]. In these applications several sensors are employed in order to achieve a more precise measurement.

Using a 5 MHz quartz crystal as external reference combined with the 32-bit precision used by the microcontroller, it allows generating square signals up to 500 kHz with a frequency resolution of 0.001 Hz.

Finally, the LIA DC output voltages V_X_ and V_Y_ are digitized by two LTC2400 24-bit ADCs from Linear Technology (Linear Technology Corporate Headquarters, Milpitas, CA, USA). These devices provide a resolution of a few microvolts operating under a single power supply of 3.3 V. This high resolution is required when applying the herein proposed LIA interface with the microcantilever-based sensors used forward in this work to prove the system capability. These devices are controlled by a serial peripheral interface (SPI) protocol, providing the corresponding readings to the microcontroller. In this way, from the digitized values of *V_X_* and *V_Y_* provided by the ADCs, the microcontroller recovers the amplitude and phase shift of the sensor signal *V_IN_* applying the equations showed in Table 1.

### 2.3. Power Supply System

The interface is powered through the 5 V line from a mini-USB connector. Additionally this port allows connecting the interface microcontroller to a computer to characterize the system and to download the collected measurements for further analysis or a more complex processing [23]. In order to reduce the inherent noise in USB power lines and to provide the required voltage levels the interface includes two voltage regulators from STMicroelectronics (STMicroelectronics, Geneva, Switzerland). On the one hand, a LD1117ADT33 provides a 3.3 V voltage level to power up the commercial devices included in the control system block. On the other hand, a 1.8 V LD1117ADT18 voltage regulator is used to bias the ASIC-based signal processing block. Two different power supplies (3.3 and 1.8 V) have been used to minimize the power consumption. However, if at a particular application, a larger SNR ratio would be needed, the herein presented design could also be implemented using a unique power supply of 3.3 V.

## 3. Experimental Verification

**Figure 6:**
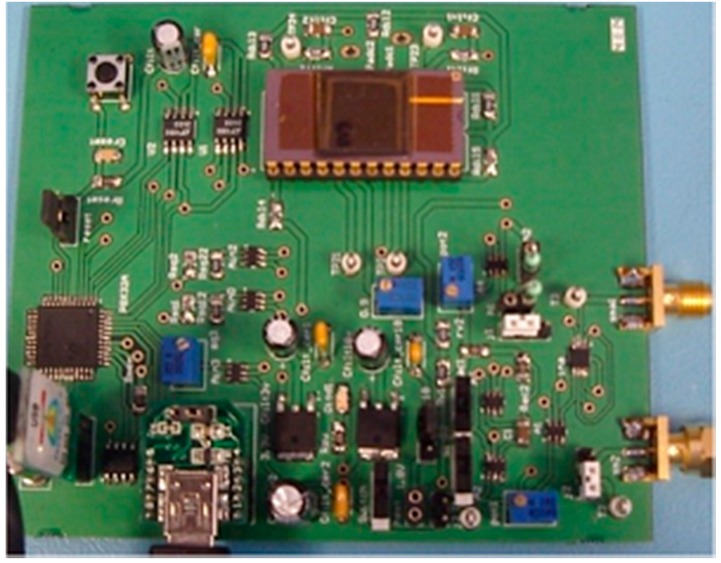
Photograph of the proposed portable electronic interface, with dimensions 85 × 99 mm^2^.

**Figure 7:**
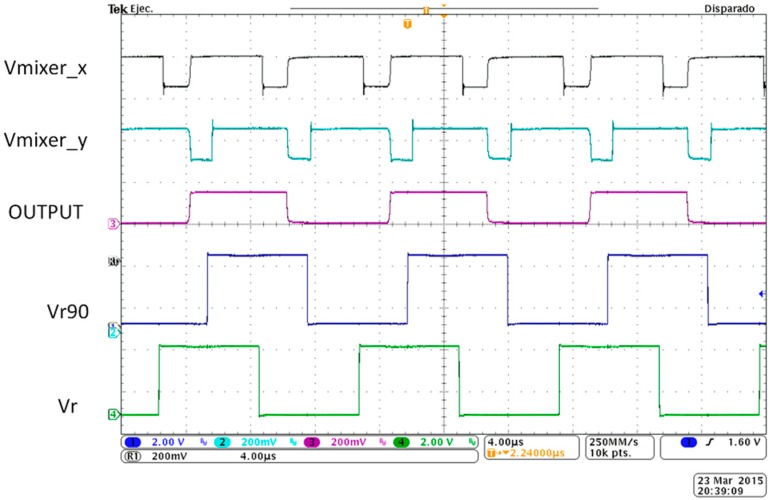
LIA signals for an input signal of 150 mV_rms_ at an operating frequency of 80 kHz. Square quadrature signals *V_r,_* (green), *V*_*r*90_ (blue), sensor signal OUTPUT (purple) and mixers signals *V_mixer_x_* (black) and *V_mixer_x_* (light blue).

Figure 6 depicts the hardware implementation of the complete LIA-based prototype. Figure 7 shows the system signals upon the introduction of a square wave signal *OUTPUT* at an operating frequency of 80 kHz with an amplitude value *A_s_* of 150 mV_rms_ and a phase shift *θ* of 70° relative to the NCO in-phase signal *V_r_*. The input signal has been generated using a Function Waveform Generator 33522A from Agilent Technologies (Agilent Technologies Inc. Headquarters, Santa Clara, CA, USA).

This signal simulates a sensor signal and it has been selected for a proper visualization of the interface operation. This section is meant to provide a full characterization of the LIA performances under several system tests. To achieve this, specific lab instrumentation have been employed to accomplish these tests as it is explained forward in this work. Firstly, the LIA response has been tested facing noise-free signals in order to prove the system linearity, error quantification and system resolution. Secondly, the behavior of the system has been proven upon the introduction of two perturbation signals: white noise and sinusoidal interferences.

### 3.1. Interface Performances

**Figure 8:**
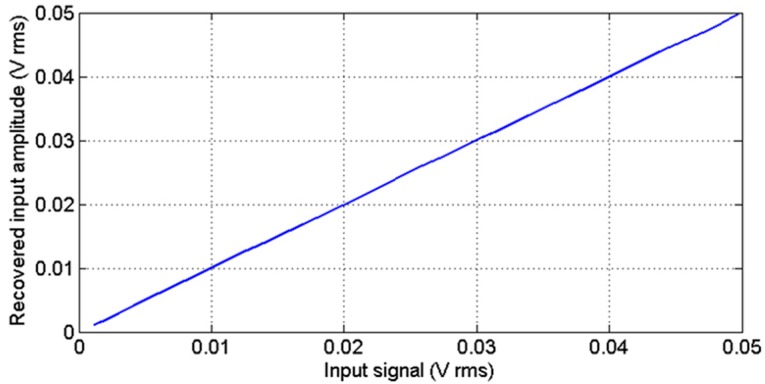
LIA experimental recovered input amplitude *vs.* input signal with different amplitude values for a 500 kHz square input.

In order to show the LIA linear response, Figure 8 shows the experimental LIA recovered values upon the introduction of a signal with variable amplitude. This test has been performed by setting unity gain (G = 1) at an operating frequency of 500 kHz that corresponds to the middle of the frequency range. The linearity error remains below 0.006% for the complete frequency range.

**Figure 9:**
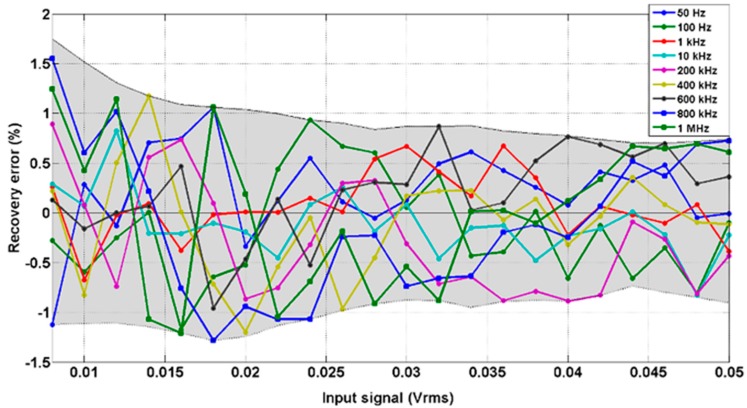
Recovery error *vs.* input amplitude (square signal) at different frequencies (G = 1). Grey area corresponds to the area between the lower and upper limits of the recovery errors.

To evaluate the recovery relative error, defined amplitude values for a square wave input signal are introduced at different operating frequencies within the frequency range. This error is calculated as the deviation between the defined input amplitude and the recovered value. Figure 9 illustrates the obtained results. As it can be shown, this error remains below 1.8% for a frequency range up to 1 MHz.

**Figure 10:**
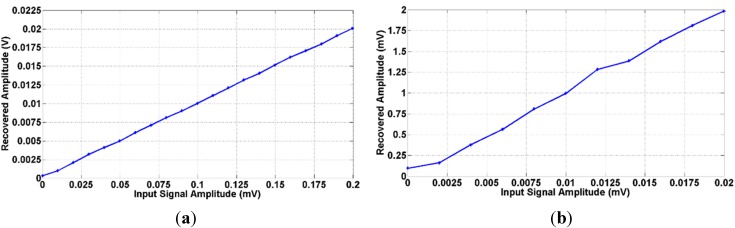
LIA experimental recovered input amplitude *vs.* input signal with amplitude values from (**a**) 1 μV to 200 μV and (**b**) 1 μV to 20 μV for a 10 kHz square input using a system gain of G = 100.

In order to establish the system resolution, a testing square signal at an operating frequency of 10 kHz and amplitude value from 1 to 200 μV is applied to the LIA with the conditioning amplifier gain set to 100 (G = 100). Figure 10 shows the amplitude recovered for different amplitude values of the input signal.

### 3.2. Noise and Interference Tests

After verifying the system ability to recover noise-free signals, the performance of the LIA-based interface has been also tested upon the introduction of noisy input signals. These tests have been carried out in the worst case by setting unity gain (G = 1). Specifically, two signal perturbations have been considered: white noise and sinusoidal interference noise at frequency close to the operating frequency and the first three odd harmonics.

A specific noise-free signal with an amplitude value of 10 mV_rms_ has been defined to perform these tests as the signal *OUTPUT*. Noise is added using a 33522A Function Waveform Generator from Agilent Technologies, which generates white noise with a bandwidth of 30 MHz, covering the bandwidth of the proposed LIA. The rms input signal has been limited to maintain significant signal-to-noise power ratio (SNR) values and to prevent saturation at 1.8 V.

The SNR is defined as:

(2) $$SNR=20\cdot \log_{10}\frac{Vrms\_signal}{Vrms\_noise}$$

where *V_rms_signal_* and *V_rms_noise_* are the effective values of the input signal and noise, respectively.

**Figure 11:**
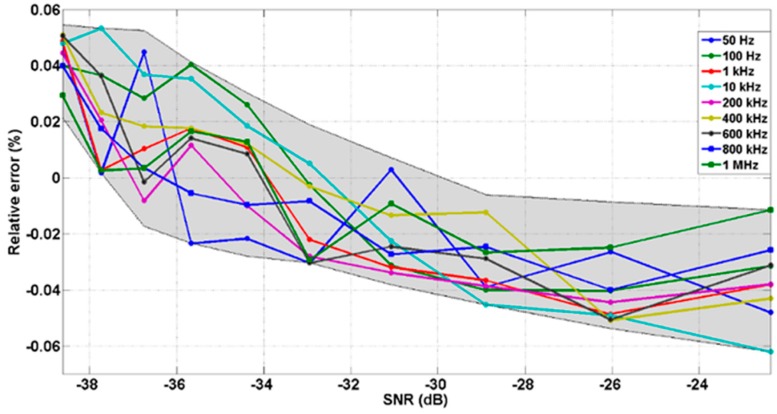
Relative error *vs.* SNR for a 10 mV_rms_ square input signal buried in white noise. Grey area corresponds to the area between the lower and upper limits of the recovery errors.

To show how noise affects to the recovered amplitude values, Figure 11 shows the relative error—defined as the deviation between the measured output signal amplitude and the recovered value once the noise has been added– upon the introduction of different noise levels. It can be highlighted that this error remains below 0.064% for all the signal frequency range up to 1 MHz, and SNR from −39 to −22 dB.

A second test has been accomplished to study the system affection due to sinusoidal interference. In case of using square signals, composed by a main frequency and the successive odd harmonics, the measurement can be mainly affected by noise frequencies close to the first three odd harmonics while this fact does not affect significantly in other spectrum frequencies.

**Figure 12:**
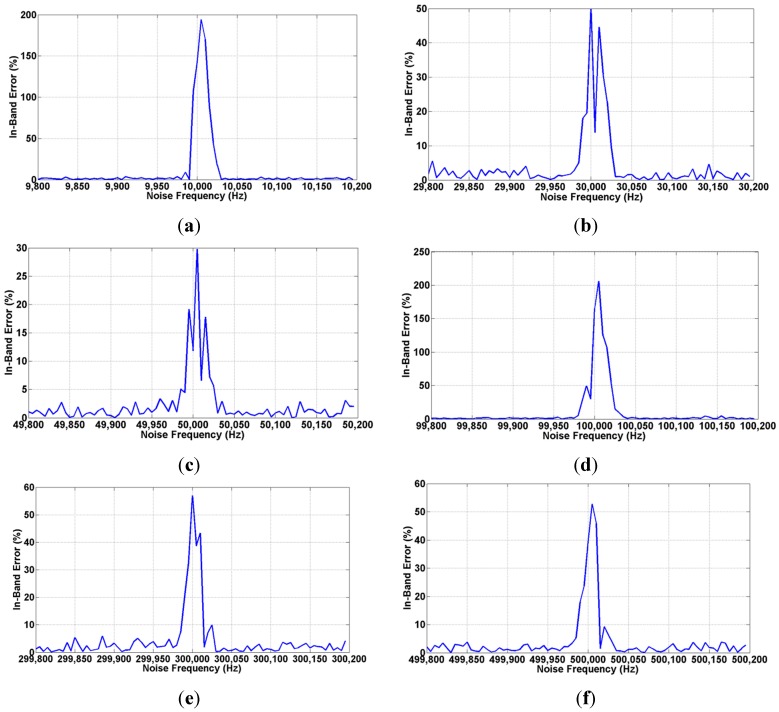
Amplitude recovery for the test signal of 10 mV_rms_no_ at an operating range of 10 kHz (**a**–**c**) and 100 kHz (**d**–**f**) in 5 Hz steps buried in −20 dB of sinusoidal interference whose frequency is around the main frequency components of the square data signals.

**Figure 13:**
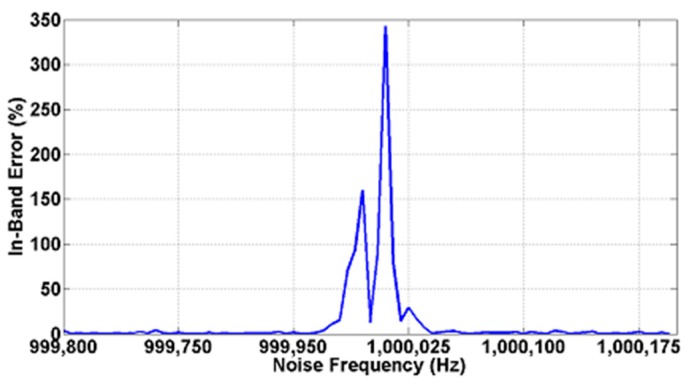
Amplitude recovery for the test signal of 10 mV_rms_ at the maximum operating frequency limit of 1 MHz buried in −20 dB of sinusoidal interference.

Figure 12 shows the in-band error (*i.e.*, relative error when the interference signals is close to an odd harmonic) when the 10 mV_rms_ square test signal presents a SNR value of −20 dB as defined in Equation (2) that concurs with the first (a, d), third (b, e) and fifth (c, f) harmonics at two operating frequencies of 10 kHz and 100 kHz in steps of 5 Hz. It is worth to highlight that there is a dead band (*i.e.*, a frequency range in which error is higher than 5%) of 40 Hz around the first two harmonics where the error quickly raises up. Table 3 summarizes these results. Figure 13 illustrates the in-band error at the operating frequency limit of 1 MHz. It is interesting that the maximum in-band error achieved can be larger if the noise levels are larger as well, however, the 40 Hz dead band span remains constant for the whole frequency range.

**Table 3:** Interference test results.

Operating Frequency	Harmonic	Max. in-Band Error (%)	Dead Band Span (Hz)
10 kHz	First	>100	35
	Third	50	40
	Fifth	30	40
100 kHz	First	>100	40
	Third	58	45
	Fifth	51	40
1 MHz	First	>100	45

These results confirm the system capability handling low and noisy signals. Table 4 summarizes the main characteristics of the presented LIA-based electronic interface and compares the proposed implementation with other CMOS implementations presented in the literature. As it can be shown, the proposed LIA allows high versatility by processing both square and sinusoidal signals, also achieving the frequency highest operation while keeping a similar power consumption respect to other prior CMOS implementations. Moreover, these performances make the herein presented interface suitable for portable gas detection applications using microcantilever-based sensors as it is explained in the next section.

**Table 4:** Performance comparison of integrated LIA-based interfaces.

Performance	Proposed LIA	[16] (2010)	[17] (2013)	[21] (2014)
**CMOS Technology (µm)**	0.18	0.35	0.35	0.18
**Supply Voltage (V)**	1.8	±1	1.8	1.8
**SNR (dB)**	−39{*ε* < 0.07%}	-	-	−42.13 {*ε* < 4.1%}
**Resolution (µV)**	1	1	-	50
**Input Signal Type**	Sine, Square	Sine	Sine	Sine
**Frequency Range (kHz)**	1000	0.077	0.25	125
**Power Consumption (mW)**	<3.5 (2 branches)	3 (1 branch)	2 (1 branch)	0.417 (1 branch)
**Integrated Area (mm^2^)**	0.048	5	-	0.013

## 4. Application to a Microcantilever-Based Resonant Sensor

In order to achieve accurate gas detection measurements at trace level, microcantilever-based resonant sensors have been revealed as a highly effective technique in security applications related to explosives detection [19,23,25]. Microcantilevers work as a mechanical resonant oscillating system. Its maximal oscillation amplitude is found at its natural frequency f_0_, which value depends on the detected amount of gas of interest. Additionally, at this frequency its characteristic phase shift is also located.

Therefore, both the characteristic amplitude and phase shift at the resonant frequency can be determined by a sweep around the theoretical resonant frequency. In this sense, the measurement of each pair of values (amplitude and phase) at each input signal frequency would provide the resonant frequency by determining the maximum amplitude value and a phase shift close to 90° and then requesting the signal frequency value provided by the microcontroller. Resonant sensors are integrated in large arrays where microbalances operate targeting different gas types at the same time. Therefore, in order to include these features in portable cyber-physical systems, complex gas detection measurements require battery-powered integrated actuation and read-out systems instead of the current available lab equipment [23,26,27].

Due to the characteristics of the proposed integrated LIA, a suitable test bench is found in its application as a signal conditioning system for microcantilever-based sensors. In this scenario, it results essential achieving a high frequency resolution in order to reach lower gas detection levels [23]. Moreover, as the sensor is encapsulated together with other sensors in a specific sensor array, the microcontroller selected in Section 2 fits perfectly these two important requirements. Similarly, a proper digitalization of the LIA signals *V_X_* and *V_Y_* must be accomplished. The acquisition time have been set to 1 s per measurement which allows using the aforementioned LTC2400 24-bit ADC for an accurate digitalization.

**Figure 14:**
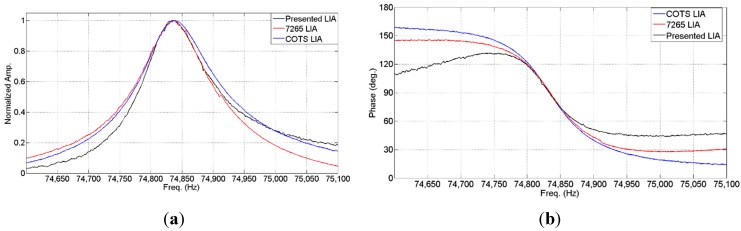
Normalized amplitude (**a**) and phase (**b**) values obtained for a frequency sweep around fundamental frequency of a microcantilever using three different recovery devices using a frequency step of 1 Hz.

Figure 14 shows the normalized amplitude (Figure 14a) and phase values (Figure 14b) obtained from a microcantilever sample [19] using three different instruments: a commercial digital lock-in amplifier 7265 from Signal Recovery (Signal Recovery, Oak Ridge, TN, USA), the portable analog LIA presented in [23] based on commercial off-the-shelf (COTS) components and the herein presented integrated LIA system, using square signal as sensor excitation and recovering the signal characteristics using the algorithm shown in Table 1.

Table 5 shows the resonant frequency determined by using each different instrument. Phase deviation out of the region of interest, the resonance region, is due to the drastically reduced response provided by the sensor that hinders a proper phase measurement, increased by the supply voltage reduction of the measurement system. Nonetheless, it is worth highlighting the similarity between these results, especially in phase tracking, since it is more sensitive than amplitude tracking for fundamental frequency determination. Table 6 summarizes the power consumption of each electronic interface and its bandwidth.

**Table 5:** Fundamental frequency measurement using different LIA implementations.

Integrated-LIA	LIA7265	COTS Prototype
f_0_ = 74,839 Hz	f_0_ = 74,838 Hz	f_0_ = 74,840 Hz

**Table 6:** Consumption comparison between different LIA implementations.

LIA Implementations	Power (W)	Bandwidth (MHz)
**LIA7265**	30	0.25
**COTS Prototype**	0.008	0.3
**Integrated LIA**	0.0035	1.0

## 5. Conclusions

This paper proposes a LIA-based low-power electronic interface capable to recover input signals up to 1 MHz buried in high noise levels. The core of the system is a novel ASIC fabricated in 1.8 V-0.18 µm CMOS. It contains a general purpose, dual-phase analog lock-in amplifier (LIA). Unlike the integrated versions reported so far, the LIA is able to operate with square input signals, so the system can benefit from the LIAs’ square algorithm in battery-operated microcontrolled systems. ASIC blocks have been carefully designed to allow high versatility and wide frequency operation, also meeting the special demands imposed by square-signal processing. In fact, experimental results for square signals buried in white noise confirm the capability of the LIA to effectively recover information from input signals buried in noise with signal-to-noise power ratios down to −39 dB with relative errors below 0.07% up to 1 MHz. The system has been also tested under interference noise showing a dead band span of 40 Hz around the first two harmonics, where the error raises up quickly. Furthermore, its operation has been tested in a specific application which involves recovering low and noisy signals like in microcantilever-based sensors. Experimental results have shown an almost identical recovery performance when compared to other LIAs based on commercial off-the-shelf (COTS) components or commercial devices.
